# A Rare but Severe Long‐Term Complication of an Implantable Chamber

**DOI:** 10.1002/ccr3.71773

**Published:** 2026-01-14

**Authors:** Raphael Grolleau, Agathe Brault, Marie‐Laure Rabilloud, Jean Michel Quedillac, Eric Rendu, Fabien Huet

**Affiliations:** ^1^ Cardiology Department Centre Hospitalier de Vannes Paris France; ^2^ Cardiology Department CHU de Rennes Paris France; ^3^ Infectious Diseases and Intensive Care CHU de Rennes Paris France; ^4^ Hepato‐Gastroenterology Department Centre Hospitalier de Vannes Paris France

**Keywords:** cardiac tamponade, catheter‐related complication, central venous access device, implantable port, long‐term device, pericardial effusion

## Abstract

Long‐term implantable ports may cause severe complications even years after placement. This case highlights the importance of verifying catheter integrity and tip position before reusing a dormant central venous access device, as delayed mechanical erosion can lead to pericardial effusion and life‐threatening cardiac tamponade.

## 1 Introduction

Cardiac tamponade is a life‐threatening condition caused by external compression of the heart due to pericardial effusion, leading to circulatory failure. The right atrium and ventricle are the first affected because of their thinner walls and lower filling pressures. As pressure increases, the right atrium collapses during systole and the right ventricle during early diastole, leading to reduced ventricular filling, diminished cardiac output, and classic signs such as hypotension, elevated jugular venous pressure, and tachypnea. Diagnosis is easily confirmed by echocardiography, and prompt pericardial drainage is the cornerstone of management. Pericardial effusion has multiple etiologies, with malignancy accounting for approximately 45% of cases. This report describes a non‐classical, device‐related cause of pericardial effusion in a patient with an implantable central venous access device (CVAD).

## 2 Case History/Examination

In 2008, a patient was hospitalized for acute necrotizing alcoholic pancreatitis. Due to severe malnutrition, parenteral nutrition was initiated using a central venous catheter with an implanted port‐a‐cath. The patient received no medical follow‐up between 2019 and 2023, during which the device was neither used, accessed, nor removed. The port remained in place without manipulation or maintenance and therefore could not have been accidentally displaced or withdrawn. In January 2024, he was diagnosed with colorectal cancer (adenocarcinoma) and liver metastases. The first course of neoadjuvant chemotherapy and anti‐EGFR was administered via the implantable port. The baseline CT scan showed the catheter's distal tip in the correct position.

## 3 Differential Diagnosis, Investigations, and Treatment

**FIGURE 1:**
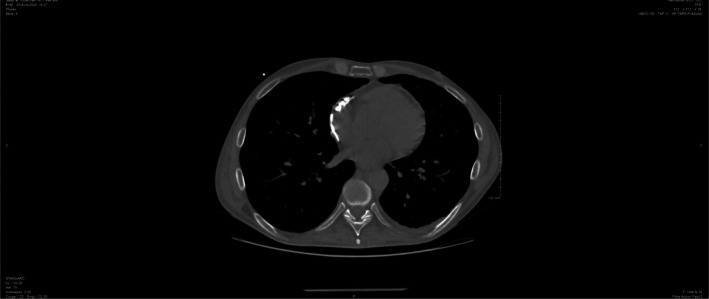
Echocardiographic section showing abundant pericardial effusion with compression of the heart CVAD.

Five days later, the patient presented to the emergency department with dyspnea and chest pain. Ultrasound revealed cardiac tamponade (Figure 1). Pericardiocentesis was performed immediately, resulting in complete resolution of the effusion, and cytological analysis showed no malignant cells. Microbiological cultures of both the pericardial fluid and the catheter tip were negative for bacterial and fungal pathogens, ruling out an infectious etiology. This finding supports a mechanical rather than infectious cause of the pericardial effusion.

**FIGURE 2:**
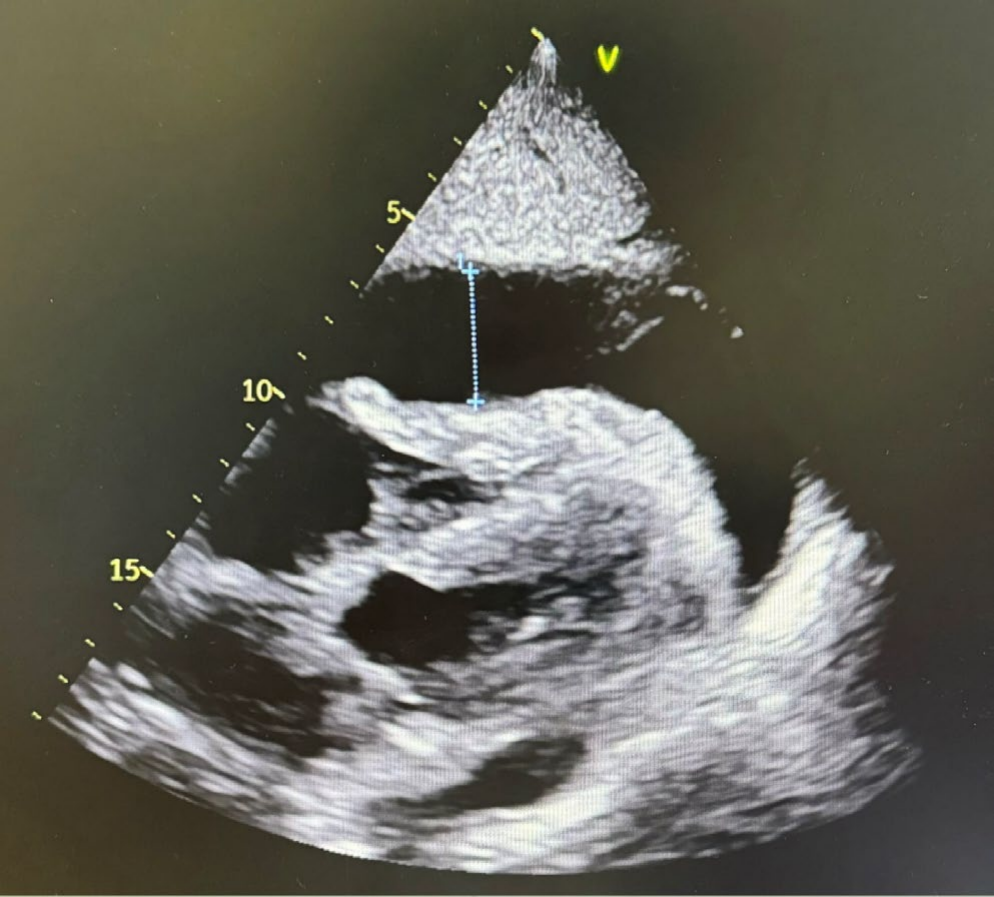
CT scan and injection of contrast into the implantable CVAD appearing in the pericardium. Confirmation of the fistulization of the catheter into the pericardium.

Three days later, a recurrence of the effusion was observed. The drain evacuated approximately 300 mL of clear fluid daily. Subsequently, iodine contrast was injected into the implanted port‐a‐cath, and a CT scan revealed direct opacification of the pericardial space due to a communication between the distal part of the catheter and the pericardium (Figure 2). Tumoral or toxic tamponade was ruled out.

The chosen treatment was surgical removal of the implantable CVAD through sternotomy, followed by suturing of the pericardium.

## 4 Conclusion and Results (Outcome and Follow‐Up)

The surgical approach was successful, and the pericardial effusion resolved with no recurrence observed at follow‐up. The patient was able to resume oncological management thereafter.

## 5 Discussion

Late post‐interventional complications due to implantable catheters are a rare etiology of pericardial effusion. These complications are rare but preventable and may occur long after catheter placement. Risk factors include prematurity, hemodialysis [1, 2], right ventricular perforation following transjugular intrahepatic portosystemic shunt (TIPS) implantation [3], and long‐term parenteral nutrition.

In this case, chronic inflammation, thrombosis, and calcification around the catheter likely contributed to a fistulous connection with the pericardium. Factors such as catheter positioning, material, and possibly prothrombotic mutations may have played a role. The unusually long delay between device implantation and the onset of pericardial effusion raises important pathophysiological considerations. Late complications of central venous catheters are often related to progressive vessel wall erosion secondary to chronic mechanical stress from the catheter tip, particularly when it abuts the venous or atrial wall. Chemotherapy‐induced vascular fragility, local thrombosis, or subclinical infection may further weaken the endothelium and adjacent tissues, predisposing to delayed perforation or fistula formation. Such mechanisms have been described in several reports of late cardiac tamponade, sometimes occurring more than a decade after device placement [4, 5].

Preventive strategies include proper catheter placement with radiological verification and long‐term surveillance. The risk of venous perforation can be minimized if the catheter tip is positioned approximately 2 cm above the pericardial reflection. Cross‐sectional imaging should always include a systematic description of the catheter path and tip location. In this case, no imaging was performed before the resumption of chemotherapy, which represents a potential contributing factor. This highlights the importance of verifying catheter integrity and tip position before administering high‐risk infusions through long‐dormant devices. Even after years of inactivity, patency should be assessed before reusing an implantable port. It is advisable to inject saline and check for reflux. A chest X‐ray with opacification may be necessary to ensure catheter permeability. There are no current guidelines regarding the risk of pericardial effusion after long‐term catheter use, and national recommendations are outdated. Nonetheless, removal of the device is usually necessary in such cases, requiring discussion with a reference surgical center [6].

This case highlights the importance of considering induced tamponade from a catheter‐related fistula in patients presenting with pericardial effusion and an implantable central venous access device.

## Author Contributions

**Raphael Grolleau:** conceptualization, data curation, writing – original draft. **Agathe Brault:** data curation. **Marie‐Laure Rabilloud:** conceptualization, validation. **Jean Michel Quedillac:** conceptualization, data curation, validation. **Eric Rendu:** conceptualization. **Fabien Huet:** conceptualization, data curation, investigation, writing – original draft.

## Funding

The authors have nothing to report.

## Consent

Written informed consent was obtained from the patient for publication of this case report and any accompanying images.

## Conflicts of Interest

The authors declare no conflicts of interest.

## Data Availability

Data sharing is not applicable to this article as no datasets were generated or analyzed during the current study.
